# Whole-Genome Sequence of Endophytic Plant Growth-Promoting *Escherichia coli* USML2

**DOI:** 10.1128/genomeA.00305-17

**Published:** 2017-05-11

**Authors:** Munirah Tharek, Kee-Shin Sim, Dzulaikha Khairuddin, Amir Hamzah Ghazali, Nazalan Najimudin

**Affiliations:** School of Biological Sciences, Universiti Sains Malaysia, Minden, Penang, Malaysia

## Abstract

*Escherichia coli* strain USML2 was originally isolated from the inner leaf tissues of surface-sterilized phytopathogenic-free oil palm (*Elaeis guineensis* Jacq.). We present here the whole-genome sequence of this plant-endophytic strain. The genome consists of a single circular chromosome of 4,502,758 bp, 4,315 predicted coding sequences, and a G+C content of 50.8%.

## GENOME ANNOUNCEMENT

*Escherichia coli* is a versatile harmless inhabitant of the gastrointestinal tract that has the ability to survive, adapt, and actively grow in extraintestinal environments, including plants (1–3). Their occurrence *in planta* offers a less competitive niche due to a gain in relative protection against adverse conditions present *ex planta* (2). In fact, interior plant tissues have become a favorable niche for bacteria capable of plant invasion due to the availability of abundant nutrients and stable environment (4). Here, we report the complete genome sequence of the plant-origin *E. coli* strain USML2, isolated from inner leaf tissues of surface-sterilized phytopathogenic-free oil palm (*Elaeis guineensis* Jacq.). Interestingly, this *E. coli* strain has the capability of *in planta* ascending migration and growth promotion of its host plant.

The whole genome was sequenced using the PacBio RSII system (Pacific Biosciences, Menlo Park, CA), with a 10-kbp single-pass SMRTbell library and C2-P4 chemistry, yielding ~74× coverage. PacBio genome data were assembled using the Hierarchical Genome Assembly Process (HGAP) version 3 (SMRT Portal version 2.3.0) workflow, including consensus polishing using the Quiver algorithm. The circular genome was validated by dot plot, BLAST, and trimmed by script. The circularized genome data were annotated using the NCBI Prokaryotic Genome Automatic Annotation Pipeline (PGAAP). Additionally, automatic annotation was enriched using the RASTtk module from Rapid Annotations using Subsystems Technology (RAST) version 2.0 (5–7).

The complete genome of *E. coli* strain USML2 is composed of a single circular chromosome of 4,502,758 bp, with an average G+C content of 50.8%. The genome annotation predicted 4,442 genes, 4,315 coding sequences (CDSs), 22 rRNAs, 86 tRNAs, 19 noncoding RNAs, and 87 pseudogenes.

In relation to its *in planta* existence, genes putatively involved in its endophytic establishment were identified; they included genes for flagellar biosynthesis (*flgLKJIHGFEDCBAMN*, *fliRQPONMLKJIHGFE*, *fliTSDCZY*, *flhDC*, *motAB*, and *flhBAE*), chemotaxis activity (*cheA*, *cheW*, *cheR*, *cheB*, *cheY*, and *cheZ*), pilus production (*pilABC* and *pilMNOPQ*) for root adhesion, and cellulose and pectin degradation enzymes for colonization and invasion. Genes involved in plant growth promotion were also recognized, including the gene for solubilization of phosphate and potassium, production of 1-aminocyclopropane-1-carboxylate (ACC) deaminase (which enhances plant growth by lowering plant ethylene levels), and those coding for enzymes in the biosynthesis of the auxinic phytohormone indole-3-acetic acid (IAA). The presence of these genes highlighted the potential of *E. coli* strain USML2 as a plant growth-promoting endophyte.

### Accession number(s).

This whole-genome shotgun project has been deposited at DDBJ/EMBL/GenBank under the accession number CP011124.
